# Association of Baseline Metabolomic Profiles With Incident Stroke and Dementia and With Imaging Markers of Cerebral Small Vessel Disease

**DOI:** 10.1212/WNL.0000000000207458

**Published:** 2023-08-01

**Authors:** Eric L. Harshfield, Hugh S. Markus

**Affiliations:** From the Stroke Research Group (E.L.H., H.S.M.), Department of Clinical Neurosciences, University of Cambridge; and Victor Phillip Dahdaleh Heart and Lung Research Institute (E.L.H., H.S.M.), University of Cambridge, United Kingdom.

## Abstract

**Background and Objectives:**

Cerebral small vessel disease is a major cause of stroke and dementia. Metabolomics can help identify novel risk factors to better understand pathogenesis and predict disease progression and severity.

**Methods:**

We analyzed baseline metabolomic profiles from 118,021 UK Biobank participants. We examined cross-sectional associations of 325 metabolites with MRI markers of small vessel disease, evaluated longitudinal associations with incident stroke and dementia, and ascertained causal relationships using Mendelian randomization.

**Results:**

In cross-sectional analyses, lower levels of apolipoproteins, free cholesterol, cholesteryl esters, fatty acids, lipoprotein particle concentrations, phospholipids, and triglycerides were associated with increased white matter microstructural damage on diffusion tensor MRI. In longitudinal analyses, lipoprotein subclasses of very large high-density lipoprotein cholesterol (HDL) were associated with an increased risk of stroke, and acetate and 3-hydroxybutyrate were associated with an increased risk of dementia. Mendelian randomization analyses identified strong evidence supporting causal relationships for many findings. A few metabolites had consistent associations across multiple analysis types. Increased total lipids in very large HDL and increased HDL particle size were associated with increased white matter damage (lower fractional anisotropy: OR: 1.44, 95% CI 1.07–1.95, and OR: 1.19, 95% CI 1.06–1.34, respectively; mean diffusivity: OR: 1.49, 95% CI 1.11–2.01, and OR: 1.24, 95% CI 1.11–1.40, respectively) and an increased risk of incident all stroke (HR: 4.04, 95% CI 2.13–7.64, and HR: 1.54, 95% CI 1.20–1.98, respectively) and ischemic stroke (HR: 3.12, 95% CI 1.53–6.38; HR: 1.37, 95% CI 1.04–1.81). Valine was associated with decreased mean diffusivity (OR: 0.51, 95% CI 0.30–0.88) and had a protective association with all-cause dementia (HR: 0.008, 95% CI 0.002–0.035). Increased levels of cholesterol in small HDL were associated with a decreased risk of incident all stroke (HR: 0.17, 95% CI 0.08–0.39) and ischemic stroke (HR: 0.19, 95% CI 0.08–0.46) and were supported by evidence of a causal association with MRI-confirmed lacunar stroke (OR: 0.96, 95% CI 0.93–0.99).

**Discussion:**

In this large-scale metabolomics study, we found multiple metabolites associated with stroke, dementia, and MRI markers of small vessel disease. Further studies may help inform the development of personalized prediction models and provide insights into mechanistic pathways and future treatment approaches.

There are 6.3 million deaths due to stroke and 1.9 million deaths due to dementia each year,^1^ and 50 million people are currently living with dementia.^2^ To develop potential new treatments, we need improved techniques for identifying novel biomarkers that can predict disease outcomes and characterize the underlying mechanisms.

While increasing evidence implicates vascular risk factors and chronic cerebrovascular disease in the pathogenesis of vascular dementia, these factors also increase the risk of neurodegenerative dementias such as Alzheimer disease (AD).^3^ Recent data have suggested treating vascular risk factors, especially hypertension, may reduce overall dementia risk.^4^ This emphasizes the potential for targeting cerebrovascular disease to reduce the burden of all types of dementia. Even with small treatment effects, this could have a major global impact.

Cerebral small vessel disease (SVD), a subtype of cerebrovascular disease that seems closely linked to dementia risk, affects the small perforating vessels within the white matter and deep gray matter nuclei. The consequences of SVD include lacunar infarcts and more chronic changes detected on MRI, including white matter hyperintensities (WMH), cerebral microbleeds, enlarged perivascular spaces, brain atrophy, and diffuse white matter damage identified using diffusion tensor imaging (DTI).^5^ SVD is a major cause of vascular dementia, and it also interacts with AD pathology to increase the probability of developing clinical dementia.^6^ Therefore, it represents an important treatment target not only to reduce vascular dementia but also to reduce the impact of neurodegenerative dementias such as AD. MRI changes indicative of SVD are common in the general population with increasing age and predict both stroke and dementia.^5^

Metabolomics, the detailed quantification of small metabolic markers in biological samples,^7^ can be used to identify novel biomarkers to diagnose and monitor disease and characterize metabolic pathways underlying disease pathogenesis.^8^ Previous metabolomics studies in cardiovascular and dementia research have been relatively small.^9,10^ Studies of patients with SVD have reported that plasma ceramide levels are associated with increased cerebral microbleeds, that numerous metabolites are associated with increased WMH volume, and that many glycerophospholipids, sphingolipids, and other metabolites are associated with MRI markers of SVD, cognition, and conversion to dementia.^11-13^ However, larger studies are required to validate and extend these findings, and these may lead to better understanding of the underlying mechanistic pathways for the development of clinical biomarkers and the identification of potential targets for intervention. In this analysis, we analyzed metabolomic profiles from 118,021 participants in the UK Biobank to characterize cross-sectional associations of 325 metabolites with MRI markers of SVD and longitudinal associations with future clinical diagnoses of stroke and dementia. We also used Mendelian randomization to evaluate whether these relationships are likely to be causal.

## Methods

### Data Source

UK Biobank is a prospective cohort study of more than 500,000 participants recruited from 22 centers throughout the United Kingdom.^14,15^ Participants were aged between 40 and 69 years at baseline assessment in 2006–2010. Participants took part in a comprehensive questionnaire and verbal interview and provided blood samples for metabolomic assays and genetic analyses.

### Metabolomics Data

Metabolic biomarker profiling of EDTA plasma samples was performed at Nightingale Health's laboratories in Finland from a random subset of 121,695 nonfasting participants, of which 118,021 were from baseline recruitment (2006–2010).^16^ High-throughput NMR spectroscopy was used to obtain 325 metabolic measures, including 168 metabolites measured in absolute levels, 81 composite biomarkers and ratios provided by UK Biobank, and a further 76 biomarker ratios that we separately derived (eTable 1, links.lww.com/WNL/C878). The platform included a wide range of biomarkers including amino acids, apolipoproteins, cholesterol and cholesteryl esters, fatty acids, phospholipids, triglycerides, and detailed measurements of lipid concentrations and compositions within 14 different lipoprotein subclasses. Details of the sample processing and biomarker quantification have been described in the UK Biobank NMR metabolomics companion document.^17^

To account for technical variation in the metabolite levels, a multistep processing procedure was applied, as previously described.^18^ In brief, for the 168 biomarkers that were measured in absolute levels, the concentrations were log transformed and adjusted in a robust linear regression model that accounted for the time between sample preparation and sample measurement, systematic differences between rows and columns on the 96-well shipment plates, and drift over time within each of the 6 spectrometers. The 81 composite biomarkers and biomarker ratios provided by UK Biobank were recalculated from their adjusted parts and an additional 76 biomarker ratios of potential biological significance were derived, resulting in a total of 325 metabolites (eTable 1, links.lww.com/WNL/C878).

### Clinical and Imaging Endpoints

In the full set of UK Biobank participants with metabolomics data at baseline (n = 118,021), we examined clinical endpoints for all stroke, ischemic stroke, intracerebral hemorrhage, all-cause dementia, AD, and vascular dementia, using algorithmically defined outcomes. Incident stroke and stroke subtypes and all-cause dementia were defined based on the earliest recorded date that the outcome occurred after baseline assessment, either from self-report at nurse interview, hospital admission records in the primary or secondary position, or death certificate records in the underlying cause or any other position. Incident AD and vascular dementia were based on hospital admission or death certificate records only. Identification of linked hospital admission and death certificate records for each endpoint was based on corresponding ICD-9 or ICD-10 codes (eTable 2, links.lww.com/WNL/C878).

In participants with metabolomics data for whom MRI had been performed (n = 10,024), we examined WMH volume and 2 DTI metrics of white matter tracts from the earliest available imaging assessment. For WMH volume, we used a UK Biobank–derived phenotype, the total volume of WMH from T1 and T2 fluid attenuated inversion recovery (FLAIR) images (measured in cubic millimeters),^19^ which we log transformed for analysis. To obtain the DTI metrics, we performed principal component analyses on UK Biobank–derived variables for 48 markers of both mean diffusivity (MD, the degree of diffusion) and fractional anisotropy (FA, the directionality of diffusion) on the FA skeleton of the diffusion brain MRI data and selected the first principal component of each as summary measures of MD and FA.^20^ We rescaled the values for the 3 imaging markers across participants using meaning centering and dividing by the SD to ensure that the effect sizes across outcomes were comparable.

### Cross-sectional and Longitudinal Analyses

We examined cross-sectional associations of MRI endpoints per 1-SD higher baseline metabolite levels using linear regression models adjusted for age at recruitment and sex. We also constructed regression models adjusted for a wide variety of possible confounders and vascular risk factors, including age at recruitment, sex, UK Biobank recruitment center, NMR spectrometer, Townsend deprivation index at recruitment (a simple census-based measure of material deprivation), taking blood pressure medications or statins at recruitment, body mass index at recruitment, smoking status at recruitment, and type 2 diabetes mellitus status (based on verbal interview, touchscreen self-report, or linked electronic health records or death register records). In addition, we conducted sensitivity analyses with further adjustment for ethnicity and APOE ε4 genotype.

In longitudinal analyses, we evaluated whether baseline metabolites predicted long-term progression to stroke and dementia and their subtypes. We constructed Cox proportional hazards regression models adjusted for age at recruitment and sex to assess the association with progression to stroke and dementia per 1-SD higher metabolite levels. These analyses were likewise conducted with adjustment for the possible confounders and vascular risk factors listed earlier.

### Mendelian Randomization Analyses

We performed 2-sample Mendelian randomization, which involves the use of genetic variants as instrumental variables in a technique that is analogous to a randomized trial,^21^ to assess whether the associations of metabolites with stroke, dementia, and imaging markers were likely to be causal. The 3 main assumptions of Mendelian randomization are that the instrumental variables (1) are associated with the exposure, (2) are not affected by confounding that would influence their association with the outcome, and (3) affect the outcome only through the exposure and do not affect any other trait with a downstream effect on the outcome.^21^ We used published genome-wide association study (GWAS) summary statistics for each metabolite measured in 115,078 participants from UK Biobank, conducted by the MRC Integrative Epidemiology Unit (IEU) at the University of Bristol.^22^ We obtained summary statistics for late-onset AD from a GWAS involving 35,274 cases and 59,163 controls from the International Genomics of Alzheimer's Project (IGAP)^23^ and for MRI-confirmed lacunar stroke from a GWAS involving 7,338 cases and 254,798 controls.^24^ Summary statistics for all stroke and ischemic stroke subtypes were obtained from individuals of European ancestry from the GIGASTROKE Consortium,^25^ consisting of 73,652 cases (62,100 cases of ischemic stroke; 10,804 cases of cardioembolic stroke; 6,399 cases of large artery stroke) and 1,234,808 controls. Summary statistics from a GWAS of participants from UK Biobank and the CHARGE Consortium were obtained for WMH (n = 42,310), MD (n = 17,467), and FA (n = 17,663).^26^ To ensure that the genetic instruments were independent, we performed clumping of each exposure dataset on all biallelic single nucleotide polymorphisms (SNPs) with a minor allele frequency >0.01 using a physical distance threshold of 10,000 kb and an *r*^2^ threshold of 0.001. We also harmonized the exposure and outcome datasets to ensure that the effect size of each SNP on an outcome and exposure were recorded relative to the same effect allele. Our primary Mendelian randomization analyses used inverse variance–weighted meta-analysis to combine the ratio estimates from each genetic variant into a single estimate of the causal effect of each metabolite on each outcome, using a random effects model to account for heterogeneity.^27^ We conducted sensitivity analyses using a variety of robust Mendelian randomization methods, which use different assumptions to make reliable causal inferences. These included MR-Egger regression, the weighted median estimator, and the simple and weighted mode-based estimators. As a further sensitivity analysis, we used the Mendelian randomization pleiotropy residual sum and outlier (MR-PRESSO) method to detect and account for outliers, which consists of 3 components: the global test, which is used to detect horizontal pleiotropy; the outlier test, which is used to correct for horizontal pleiotropy through outlier removal; and the distortion test, which is used to test for significant differences in the causal estimates before and after correction for outliers.^28^

All statistical analyses were conducted using R version 4.1.1 (R Core Team, 2021), and results were presented using 2-sided *p* values and 95% CIs. We used a false discovery rate (FDR) threshold of *q* < 0.05 to account for multiple testing comparisons when identifying significant associations for each outcome measure. For comparison purposes, we also calculated whether the *p* values were significant when applying a more conservative Bonferroni correction adjusted for the number of metabolites analyzed. Mendelian randomization analyses were conducted using the TwoSampleMR package version 0.5.4 and the MR-PRESSO package version 1.0.

### Standard Protocol Approvals, Registrations, and Patient Consents

All UK Biobank participants provided informed consent as part of the recruitment process to the use of their anonymized data and samples for any health-related research, to be recontacted for further substudies, and for UK Biobank to access their electronic health records. UK Biobank has approval from the North West Multicenter Research Ethics Committee as a Research Tissue Bank. This research was conducted using UK Biobank under application number 36509.

### Data Availability

Data supporting the findings of this study are available within the article and its Supplemental Materials. The original metabolomics data and phenotypic information can be accessed by approved researchers through application to UK Biobank (ukbiobank.ac.uk/enable-your-research). The summary statistics from the UK Biobank metabolomics GWAS can be accessed from the MRC IEU OpenGWAS Project (gwas.mrcieu.ac.uk/), the summary statistics for AD can be obtained from the IGAP (niagads.org/datasets/ng00075), and the summary statistics for stroke from the GIGASTROKE Consortium can be obtained from the GWAS Catalog (ebi.ac.uk/gwas/; study accession numbers GCST90104534–GCST90104563).

## Results

### Participant Characteristics

We analyzed data from 118,021 participants from UK Biobank with metabolomics measurements, of whom 54% were female and 95% were White, with a mean age of 56.5 (SD: 8.1) years (Table 1). The median follow-up time was 13.1 years (5th-95th percentile, 11.8–14.4 years). The characteristics of UK Biobank participants whose blood samples were randomly selected for the metabolomics assay to be performed were broadly comparable with those who were not selected (eTable 3, links.lww.com/WNL/C878).

**Table 1:**
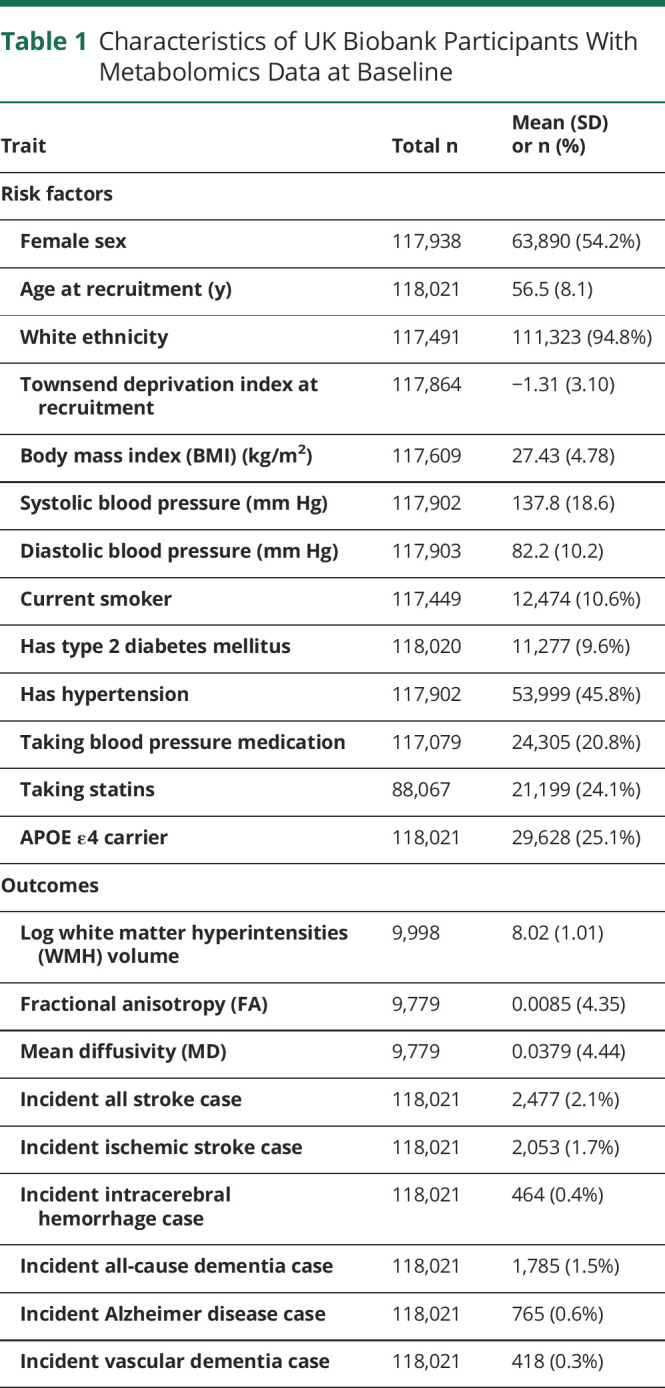
Characteristics of UK Biobank Participants With Metabolomics Data at Baseline

### Cross-sectional Associations With Imaging Parameters

We analyzed the association of 325 baseline metabolic measures with MRI markers at the first imaging assessment in cross-sectional analyses adjusted for age at recruitment and sex (eFigure 1, links.lww.com/WNL/C877, eTable 4, links.lww.com/WNL/C878). Lower levels of apolipoproteins, cholesterol, free cholesterol, cholesteryl esters, fatty acids, lipoprotein particle concentrations, phospholipids, triglycerides, and total lipids and higher levels of amino acids, glucose, and glycoprotein acetyls (GlycA; an inflammatory marker) were associated with increased white matter microstructural damage on DTI, as indicated by higher WMH and MD and lower FA.

**Figure 1:**
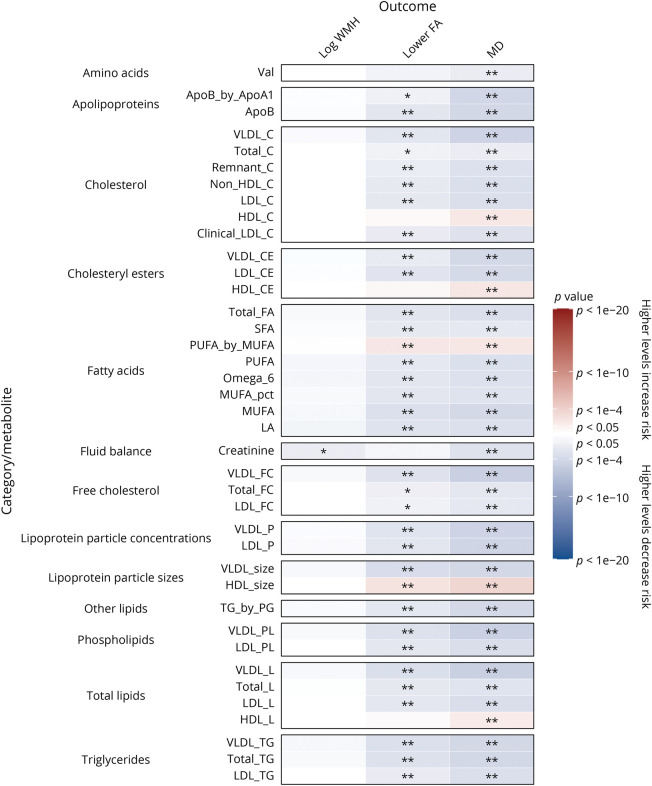
Association of MRI Markers at First Imaging Visit Per 1-SD Higher Baseline Levels of Lipids and Other Metabolites With Adjustment for Possible Confounders and Vascular Risk Factors Beta estimates and *p* values were obtained from linear or logistic regression models adjusted for age at recruitment, sex, UK Biobank recruitment center, Townsend deprivation index at recruitment, whether the person was taking blood pressure medication or statins, body mass index, smoking status, and type 2 diabetes mellitus status. Colors show magnitude and direction of *p* value for association of metabolite with each outcome (red indicates a positive association, and blue indicates an inverse association). Asterisks indicate significance: *p < 0.05; **FDR *q* < 0.05. FDR = false discovery rate.

**Figure 2:**
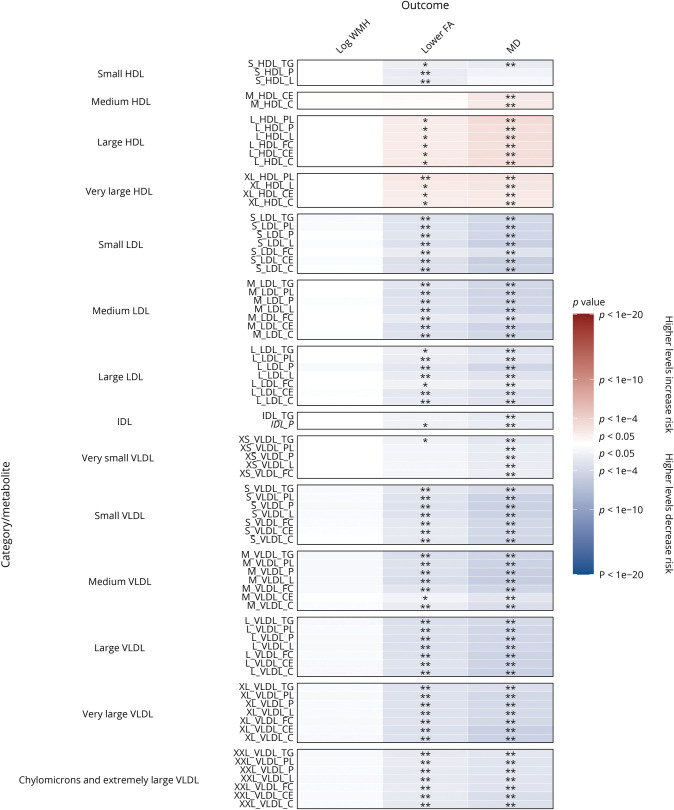
Association of MRI Markers at First Imaging Visit Per 1-SD Higher Baseline Levels of Lipoprotein Subclasses With Adjustment for Possible Confounders and Vascular Risk Factors Beta estimates and *p* values were obtained from linear or logistic regression models adjusted for age at recruitment, sex, UK Biobank recruitment center, Townsend deprivation index at recruitment, whether the person was taking blood pressure medication or statins, body mass index, smoking status, and type 2 diabetes mellitus status. Colors show magnitude and direction of *p* value for association of metabolite with each outcome (red indicates a positive association, and blue indicates an inverse association). Asterisks indicate significance: *p < 0.05; **FDR *q* < 0.05. FDR = false discovery rate; HDL = high-density lipoprotein cholesterol; IDL = intermediate-density lipoprotein cholesterol; LDL = low-density lipoprotein cholesterol; VLDL = very low-density lipoprotein cholesterol.

**Figure 3:**
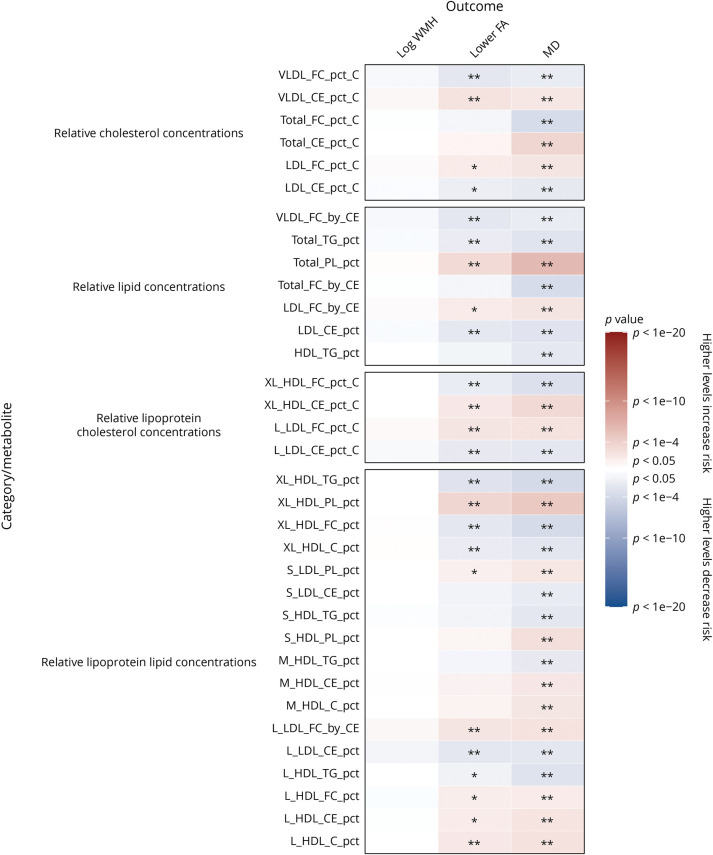
Association of MRI Markers at First Imaging Visit Per 1-SD Higher Baseline Levels of Relative Lipid, Lipoprotein, and Cholesterol Concentrations With Adjustment for Possible Confounders and Vascular Risk Factors Beta estimates and *p* values were obtained from linear or logistic regression models adjusted for age at recruitment, sex, UK Biobank recruitment center, Townsend deprivation index at recruitment, whether the person was taking blood pressure medication or statins, body mass index, smoking status, and type 2 diabetes mellitus status. Colors show magnitude and direction of *p* value for association of metabolite with each outcome (red indicates a positive association, and blue indicates an inverse association). Asterisks indicate significance: *p < 0.05; **FDR *q* < 0.05. FDR = false discovery rate.

In cross-sectional analyses adjusted for possible confounders and additional vascular risk factors, most metabolites remained significantly associated with FA and MD (Figures 1–3, and eTable 5, links.lww.com/WNL/C878). Lower levels of apolipoproteins, cholesterol, free cholesterol, cholesteryl esters, fatty acids, lipoprotein particle concentrations, phospholipids, triglycerides, and total lipids were associated with higher MD and lower FA on DTI, indicating increased white matter microstructural damage. However, increased total lipids in very large HDL (XL_HDL_L) and increased HDL particle size (HDL_size) were associated with lower FA (OR: 1.44, 95% CI 1.07–1.95, and OR: 1.19, 95% CI 1.06–1.34, respectively) and higher MD (OR: 1.49, 95% CI 1.11–2.01, and OR: 1.24, 95% CI 1.11–1.40, respectively). Higher levels of high-density lipoprotein cholesterol (HCL_C) and lipoproteins within large HDL and very large HDL were also associated with increased white matter microstructural damage.

### Longitudinal Analyses of Stroke and Dementia

In time-to-event analyses adjusted for age and sex, which took into account long-term follow-up (eFigure 2, links.lww.com/WNL/C877, eTable 6, links.lww.com/WNL/C878), higher levels of the ratio of free cholesterol to cholesteryl esters in very small very low-density lipoprotein cholesterol (VLDL) (XS_VLDL_FC_by_CE) and higher levels of GlycA were associated with an increased risk of all stroke. Conversely, higher levels of docosahexaenoic acid (DHA) and several lipoprotein subclasses containing cholesterol, free cholesterol, and cholesteryl esters were associated with a decreased risk of all stroke. Higher levels of glucose and the percentage of phospholipids to total lipids in very small VLDL (XS_VLDL_PL_pct) were associated with an increased risk of all-cause dementia, while a wide range of cholesterol, cholesteryl esters, free cholesterol, apolipoprotein B, and lipoprotein subclasses were associated with a decreased risk of all-cause dementia.

**Figure 4:**
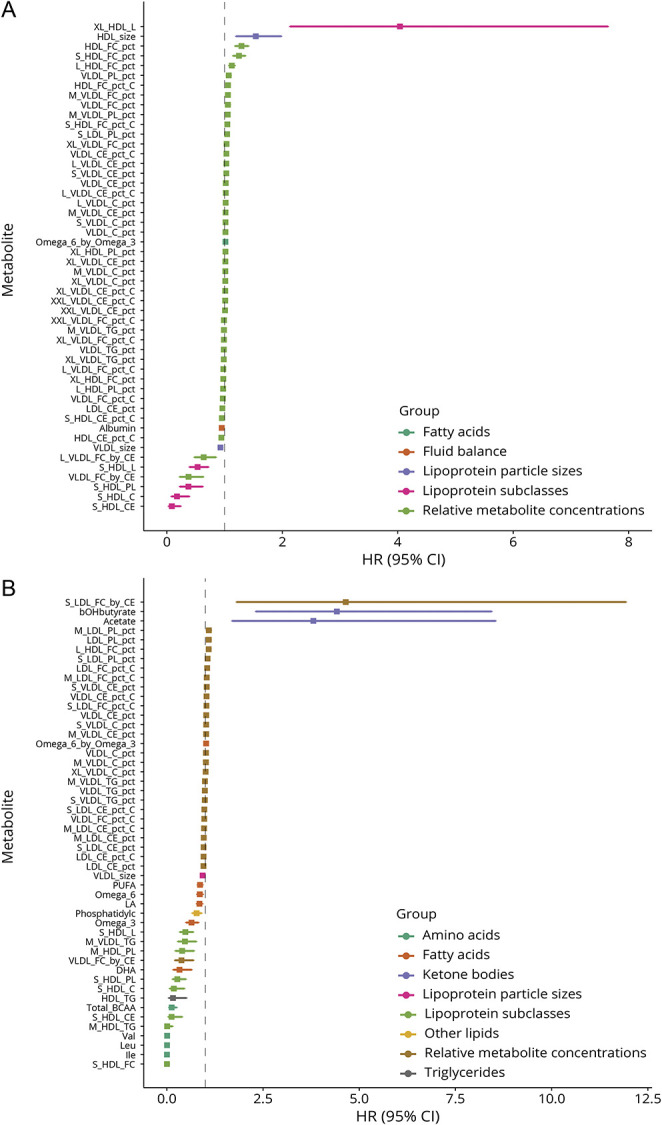
Adjusted Hazard Ratios for Incident All Stroke and All-Cause Dementia Per 1-SD Higher Baseline Metabolite Levels With Adjustment for Possible Confounders and Vascular Risk Factors (A) All stroke. (B) All-cause dementia. Analyses were adjusted for age at recruitment, sex, UK Biobank recruitment center, Townsend deprivation index at recruitment, whether the person was taking blood pressure medication or statins, body mass index, smoking status, and type 2 diabetes mellitus status. Filled squares indicate associations significant at FDR *q* < 0.05. FDR = false discovery rate.

In longitudinal analyses adjusted for potential confounders and vascular risk factors (Figure 4, eTable 7, links.lww.com/WNL/C878), total lipids in very large HDL (XL_HDL_L) were most strongly associated with all stroke (HR: 4.04, 95% CI 2.13–7.64), followed by HDL particle size (HR: 1.54, 95% CI 1.20–1.98), while cholesterol, cholesteryl esters, phospholipids, and total lipids in small HDL (S_HDL_C, S_HDL_CE, S_HDL_PL, and S_HDL_L, respectively) had inverse associations with all stroke. Acetate, 3-hydroxybutyrate (bOHbutyrate), and the ratio of free cholesterol to cholesteryl esters in small low-density lipoprotein cholesterol (LDL) (S_LDL_FC_by_CE) were associated with an increased risk of all-cause dementia. Meanwhile, omega-3 fatty acids, DHA, leucine, isoleucine, valine, the total concentration of branched-chain amino acids (Total_BCAA), and several lipoprotein subclasses were associated with a decreased risk of all-cause dementia.

Very few metabolites were significantly associated with stroke and dementia subtypes in analyses adjusted for potential confounders and vascular risk factors (eFigure 3, links.lww.com/WNL/C877, eTable 7, links.lww.com/WNL/C878). The main exceptions were that decreased levels of lipoprotein subclasses containing cholesterol, cholesteryl esters, phospholipids, and total lipids in small HDL and increased levels of total lipids in very large HDL (XL_HDL_L) and the proportion of free cholesterol to total lipids in HDL, small HDL, and large HDL (HDL_FC_pct, S_HDL_FC_pct, and L_HDL_FC_pct, respectively) were associated with an increased risk of ischemic stroke. In addition, increased levels of valine, leucine, and total branched-chain amino acids (Total_BCAA) were associated with a decreased risk of AD. In sensitivity analyses with further adjustment for ethnicity and APOE ε4 genotype, the results for all stroke and all-cause dementia were very similar and broadly comparable (eFigure 4, links.lww.com/WNL/C877, eTable 8, links.lww.com/WNL/C878).

### Mendelian Randomization Analyses

**Figure 5:**
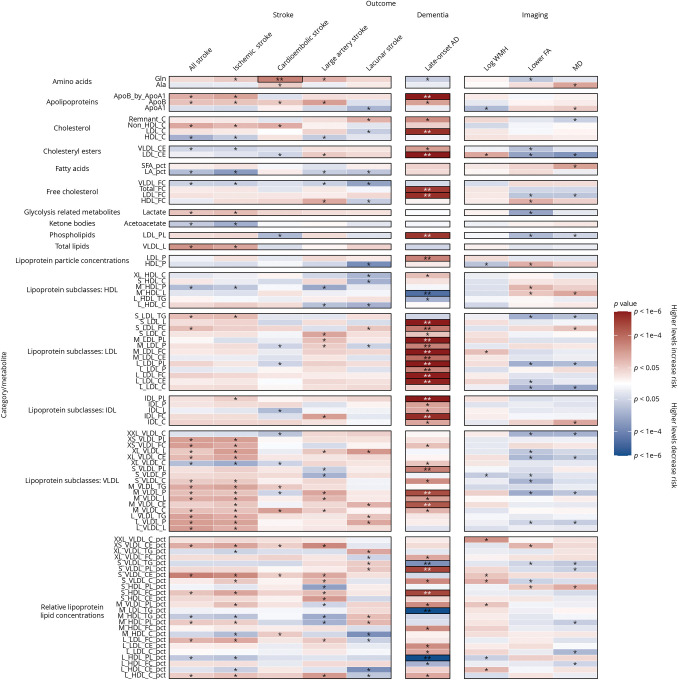
Mendelian Randomization Results Showing Causal Estimates for Association of Genetically Determined Metabolite Levels With Stroke, Dementia, and MRI Markers Colors show the magnitude and direction of the *p* value of association for the estimate of the causal effect using Mendelian randomization (the method that yielded the most significant *p* value is shown, where red indicates a positive association and blue indicates an inverse association). Asterisks indicate significance: *p < 0.05; **FDR *q* < 0.05. A thick black border delineates associations that are also significant using the MR-Egger approach, indicating putative causal relationships that are more robust to pleiotropy. FDR = false discovery rate.

Our Mendelian randomization analyses showed that genetically elevated levels of cholesteryl esters in very large VLDL and total lipids in small VLDL were associated with an increased risk of ischemic stroke, and genetically lowered levels of the concentration of HDL particles were associated with an increased risk of lacunar stroke (Figure 5, eTables 9 and 10, links.lww.com/WNL/C878). Furthermore, genetically elevated levels of LDL within cholesterol, cholesteryl esters, free cholesterol, and phospholipids, and lipoprotein subclasses of LDL, VLDL, and intermediate-density lipoprotein cholesterol (IDL) and genetically lower levels of total lipids in medium HDL were associated with an increased risk of late-onset AD. The MR-PRESSO global test revealed the presence of horizontal pleiotropy that was statistically significant (FDR *q* < 0.05) in 70.6% (n = 1,741) of the 2,465 tests (eTable 11). The MR-PRESSO outlier test identified false-positive relationships in 2.8% of putatively causal relationships (n = 1,295 of 46,857 total tests) using a threshold of *p* < 0.05 (eTable 12). The MR-PRESSO distortion test revealed that horizontal pleiotropy introduced distortions in the causal estimates with a median of 24.8% (5th–95th percentile, −356% to 1,758%) (eFigure 5, links.lww.com/WNL/C877, eTable 13). The MR-PRESSO outlier-corrected results are summarized in eTable 14.

## Discussion

In this large-scale metabolomics study of 118,021 individuals, we identified 289 metabolic traits significantly associated with MRI markers of SVD and future risk of stroke and dementia in a large population-based cohort. We found that lower levels of apolipoproteins, cholesterol, free cholesterol, cholesteryl esters, fatty acids, lipoprotein particle concentrations, phospholipids, triglycerides, and total lipids were associated with increased white matter microstructural damage on DTI. In addition, lower levels of amino acids and fatty acids and higher levels of ketone bodies were associated with an increased risk of all-cause dementia. Increased levels of lipoprotein subclasses within large HDL and very large HDL were associated with increased white matter microstructural damage and an increased risk of all stroke and all-cause dementia, whereas lipoprotein subclasses within small and medium HDL were associated with decreased white matter microstructural damage and a decreased risk of all stroke and all-cause dementia. We did not observe associations of metabolites with stroke and dementia subtypes, but the analyses may have lacked sufficient power due to the fewer number of cases for these outcomes.

A few metabolites had consistent associations across multiple types of analyses (cross-sectional, longitudinal, and/or Mendelian randomization), enabling a greater level of confidence in the importance of the findings. Increased total lipids in very large HDL and increased HDL particle size at baseline were significantly associated with increased white matter microstructural damage on DTI at the imaging assessment and significantly associated with an increased risk of incident all stroke and ischemic stroke, but there was not sufficient evidence to support a causal relationship. Valine was associated with decreased MD and had a protective association with all-cause dementia, but it also lacked evidence of causality. Cholesterol in small HDL, however, had an inverse association with incident all stroke and ischemic stroke that was supported by evidence of a causal association with MRI-confirmed lacunar stroke in the Mendelian randomization analyses. We lacked information on ischemic stroke subtypes in the observational data, but the association with cholesterol in small HDL seems to be specific to lacunar stroke.

Another notable finding was that metabolites were much more strongly associated with DTI markers than with WMH lesion volume. This is consistent with previous studies showing DTI is more sensitive to white matter damage in SVD and that DTI parameters correlate with cognition more strongly than WMH lesion volume.^29-31^

Although there is a well-established inverse association of plasma HDL cholesterol levels with coronary heart disease, stroke, and vascular brain damage, whether these relationships are causal has remained uncertain.^32-39^ This study provides new insights into these relationships by demonstrating that the direction and magnitude of the association of HDL with stroke and dementia and their subtypes depend on the size of the lipoprotein subclasses within HDL.

Many of the cross-sectional associations of metabolites with white matter microstructural damage were not in the direction that would normally be expected. Lower levels of LDL, VLDL, and total cholesterol; cholesteryl esters, free cholesterol, and triglycerides in LDL and VLDL; and cholesterol, free cholesterol, cholesteryl esters, triglycerides, and phospholipids within small, medium, and large LDL, within IDL, and within very small, small, medium, large, very large, and extremely large VLDL were associated with lower FA and increased MD. However, the associations of these metabolites with stroke were not statistically significant, and there was limited evidence from the Mendelian randomization analyses that these associations may be causal.

Our metabolite associations for all-cause dementia, AD, and vascular dementia confirm the findings reported in a recent preprint.^40^ However, we analyzed an expanded set of metabolites by including 76 additionally derived biomarkers of potential biological significance, and we evaluated associations with a wider range of endpoints including all stroke, ischemic stroke, intracerebral hemorrhage, and DTI markers.

Metabolic dysfunction is believed to play an important role in cognitive decline and AD progression.^41^ In AD, the brain loses its ability to effectively use glucose, the primary energy substrate in the brain, so ketone bodies may be an effective alternative energy substrate.^42^ Previous studies have shown that elevated levels of 3-hydroxybutyrate, a ketone body also known as β-hydroxybutyrate, are associated with improved cognition and a reduced risk of AD.^42,43^ However, our study showed that elevated levels of 3-hydroxybutyrate were associated with an increased risk of all-cause dementia and AD (although the association with AD was not statistically significant after correction for multiple testing), in concordance with the recent preprint.^40^

Mendelian randomization analyses identified evidence of causal associations of multiple metabolites with stroke, dementia, and MRI markers. However, we detected substantial horizontal pleiotropy, meaning that some genetic variants were associated with multiple metabolites on different pathways,^21^ which is unsurprising due to strong correlations between metabolites. MR-PRESSO indicated the presence of significant horizontal pleiotropy in 68% of the metabolite-outcome associations tested, which introduced substantial distortions in the causal estimates. This makes it difficult to assess whether the identified causal relationships are genuine or spurious and introduces challenges in drawing meaningful causal inferences, so the findings should be cautiously interpreted.

There are several important clinical implications arising from these findings. First, this research provides new insights into the metabolic pathways underlying stroke and dementia. This could be used to help inform dietary interventions or the development of novel therapies because modifying levels of specific metabolites might help reduce the risk of vascular-related conditions. Second, clinicians might be able to use these findings to predict which patients are most likely to develop stroke and dementia and offer personalized treatment plans.

This study has several strengths. First, we had metabolomics data on nearly 120,000 individuals, which greatly increased the power to detect statistically significant associations. Second, a fully automated, comprehensive spectrum analysis was used to measure the metabolites under strict quality control, which increased the accuracy and validity of the findings. Third, the prospective study design and long follow-up period, with metabolites that were measured before disease onset, was particularly valuable for evaluating their associations with risk of incident stroke and dementia. Fourth, we conducted sensitivity analyses to assess whether potential confounders and vascular risk factors, including APOE ε4 genotype, affected the identified associations.

There are also limitations. First, we conducted numerous statistical tests. Although we corrected for multiple testing by applying an FDR threshold to reduce the possibility of identifying false positives, this may have inadvertently led us to discount some biologically and clinically meaningful associations that did not reach the threshold for statistical significance. Second, although the sample size was large, it is not representative of the overall UK population.^44^ Third, we did not have endpoints for ischemic stroke subtypes in UK Biobank (e.g., large artery, cardioembolic, and lacunar stroke), so we were unable to examine direct observational associations of metabolites with lacunar stroke. However, our Mendelian randomization analyses helped mitigate this by identifying evidence that genetically elevated levels of metabolites are associated with an increased risk of lacunar stroke. Fourth, we obtained genome-wide association summary statistics with late-onset AD but not with all-cause dementia and vascular dementia, which limited the scope of our assessment of causal relationships of metabolites with dementia. Finally, a limitation of our 2-sample Mendelian randomization analyses is that UK Biobank was not only used for the genetic associations with the metabolites but also contributed significantly to the datasets used for the genetic associations with the outcomes, which may contribute to bias due to overfitting, although owing to the large sample sizes of the respective studies, the bias due to sample overlap is expected to be very small.^45^

In conclusion, our study provides evidence supporting the association of a wide range of metabolites with stroke, dementia, and MRI markers of SVD. Although further research is needed, these findings could help inform the development of personalized prediction models and novel treatment approaches.

## Glossary

AD: Alzheimer disease
DHA: docosahexaenoic acid
DTI: diffusion tensor imaging
FA: fractional anisotropy
FDR: false discovery rate
GlycA: glycoprotein acetyls
ICD-9: *International Classification of Diseases–9*
IEU: Integrative Epidemiology Unit
IGAP: International Genomics of Alzheimer's Project
MD: mean diffusivity
MR-PRESSO: Mendelian randomization pleiotropy residual sum and outlier
SVD: small vessel disease
WMH: white matter hyperintensities
